# Tracheal Diverticulum Following Paratracheal Hypofractionated Radiotherapy in the Setting of Prior and Subsequent Bevacizumab

**DOI:** 10.7759/cureus.578

**Published:** 2016-04-19

**Authors:** Aadel A Chaudhuri, Jie Jane Chen, Justin N Carter, Michael S Binkley, Kiran A Kumar, Sara A Dudley, Arthur W Sung, Billy W Loo Jr.

**Affiliations:** 1 Department of Radiation Oncology, Stanford University School of Medicine; 2 Department of Pulmonary and Critical Care Medicine, Stanford University School of Medicine; 3 Stanford Cancer Institute, Stanford University School of Medicine

**Keywords:** tracheal diverticulum, bevacizumab, vegf inhibitor, hypofractionated conformal radiotherapy, volumetric modulated arc therapy (vmat), oligometastatic cancer

## Abstract

We present the case of a 63-year-old woman with limited metastatic colorectal cancer to the lungs and liver treated with FOLFIRI-bevacizumab, followed by consolidative hypofractionated radiotherapy to right paratracheal metastatic lymphadenopathy. We treated the right paratracheal site with 60 Gy in 15 fractions (70 Gy equivalent dose in 2 Gy fractions). The patient tolerated the treatment well, and six months later started a five-month course of FOLFIRI-bevacizumab for new metastatic disease. She presented to our clinic six months after completing this, complaining of productive cough with scant hemoptysis, and was found to have localized tracheal wall breakdown and diverticulum in the region of prior high-dose radiation therapy, threatening to progress to catastrophic tracheovascular fistula. This was successfully repaired surgically after a lack of response to conservative measures. We urge caution in treating patients with vascular endothelial growth factor (VEGF) inhibitors in the setting of hypofractionated radiotherapy involving the mucosa of tubular organs, even when these treatments are separated by months. Though data is limited as to the impact of sequence, this may be particularly an issue when VEGF inhibitors follow prior radiotherapy.

## Introduction

Bevacizumab is a monoclonal antibody that antagonizes the pro-angiogenic vascular endothelial growth factor (VEGF), and can be a highly effective agent for treating metastatic cancer; it inhibits tumor growth by limiting its blood supply. Bevacizumab has also been shown to be effective when given concurrently and adjuvantly with radiotherapy in the treatment of glioblastoma multiforme [1]. However, thoracic/mediastinal radiation therapy and bevacizumab have been associated with complications such as tracheoesophageal fistula [2-6].

**Figure 1:**
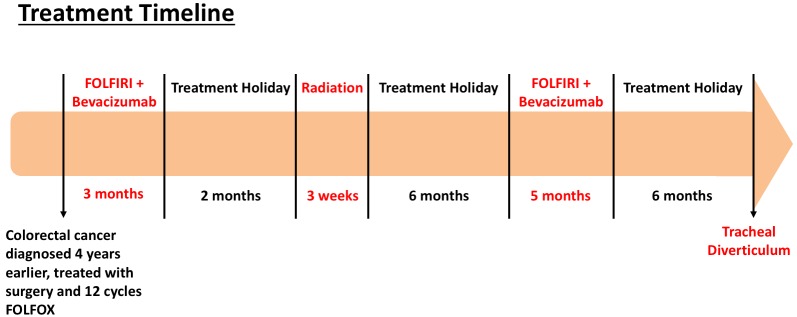
Treatment timeline for patient with metastatic colorectal cancer.

We report a case of a patient with oligometastatic colorectal cancer who was treated with FOLFIRI-bevacizumab followed two months later by hypofractionated radiotherapy, and six months later treated with FOLFIRI-bevacizumab again. This patient unfortunately experienced a tracheal diverticulum 17 months after radiotherapy and six months after her second course of FOLFIRI-bevacizumab (Figure 1). Informed consent was obtained from the patient for this study.

## Case presentation

**Figure 2:**
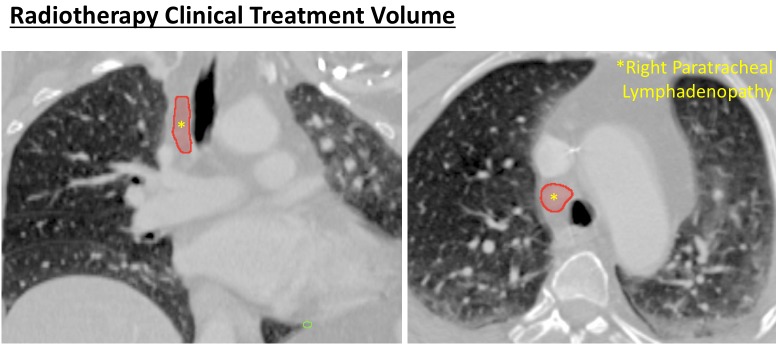
Pre-radiotherapy CT scan showing right paratracheal clinical treatment volume (CTV). CTV is outlined in red and indicated with yellow asterisk.

**Figure 3:**
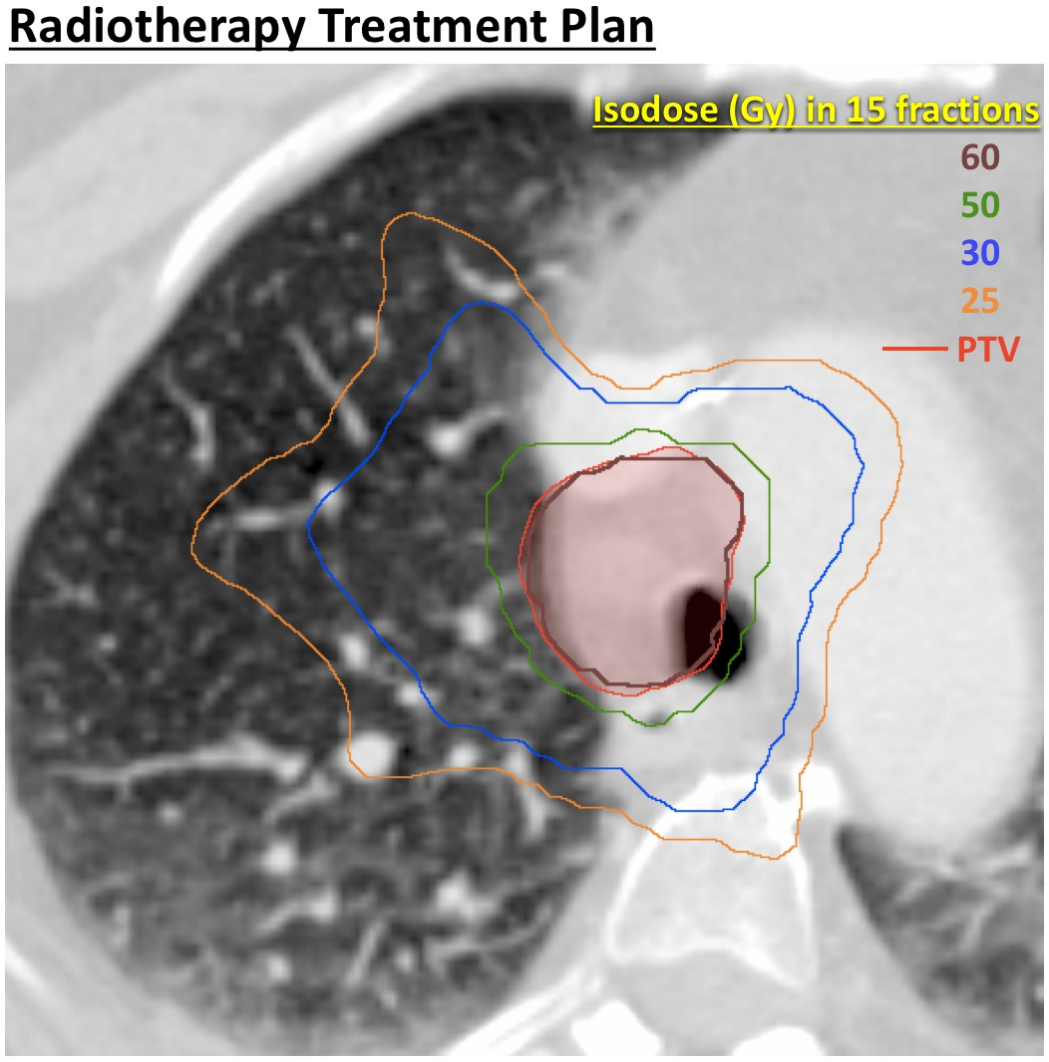
Radiation treatment plan of right paratracheal lymphadenopathy with 60 Gy in 15 fractions. Radiotherapy dose is depicted as isodose levels. Planning target volume (PTV) is outlined in red.

A 63-year-old nonsmoking woman with metastatic colorectal cancer to the lungs and liver, diagnosed four years previously and treated at that time with surgery and 12 cycles of FOLFOX, presented to our clinic with oligoprogression of right paratracheal metastatic lymphadenopathy. We initially recommended optimization of her systemic therapy. She underwent treatment with three months of FOLFIRI-bevacizumab followed by a two-month treatment holiday, and presented to our clinic again with decreased right paratracheal lymphadenopathy and no new metastatic disease. Considering her limited disease burden and long progression-free interval, we offered definitive dose hypofractionated radiotherapy as consolidation, and treated her with 60 Gy in 15 fractions with highly conformal volumetric modulated arc therapy (VMAT) (Figures 2-3). The patient tolerated the treatment well. However six months later she developed new metastatic disease in the lung and abdomen and recommenced FOLFIRI-bevacizumab for five months.

**Figure 4:**
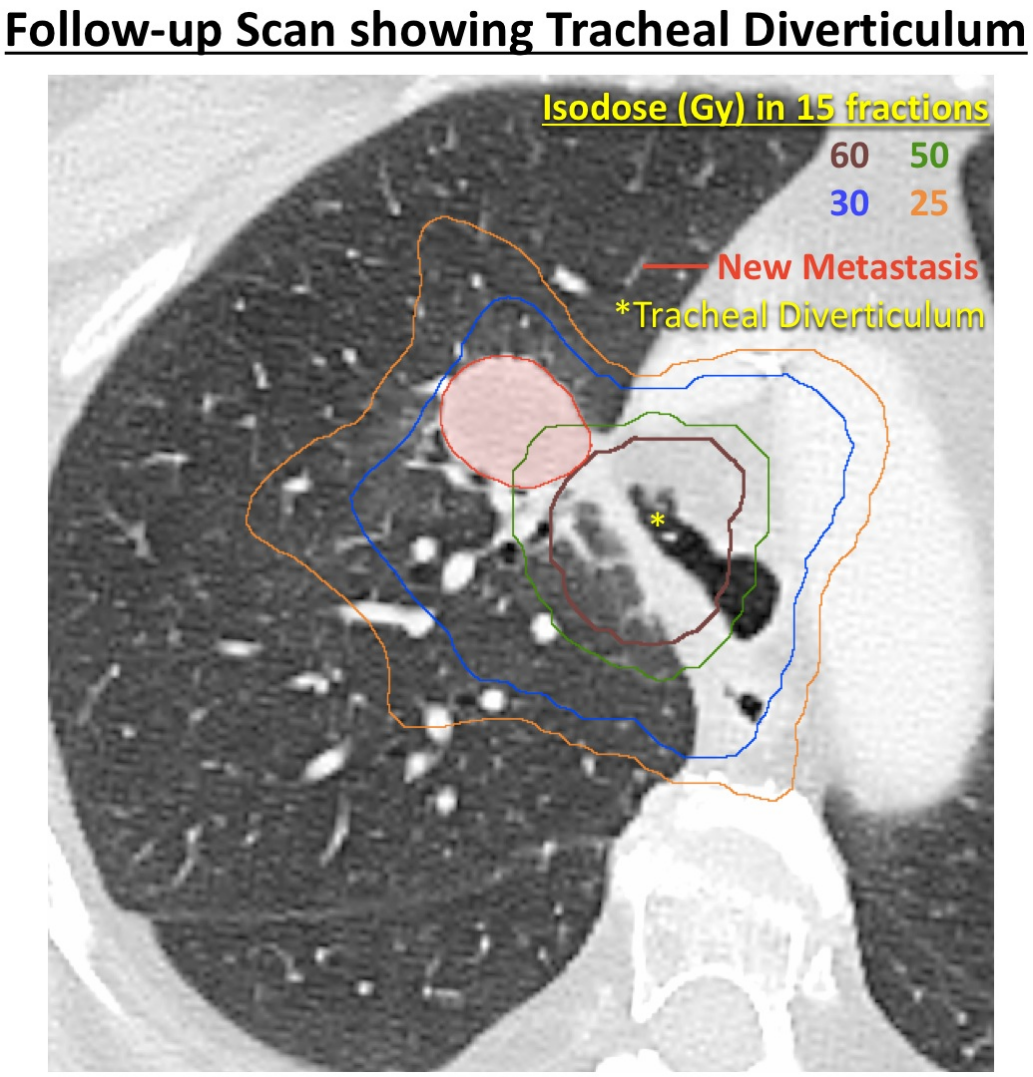
Follow-up CT scan showing tracheal diverticulum in the region of prior high-dose radiotherapy. Prior radiotherapy dose is depicted as isodose levels. A new metastatic nodule is outlined in red.

**Figure 5:**
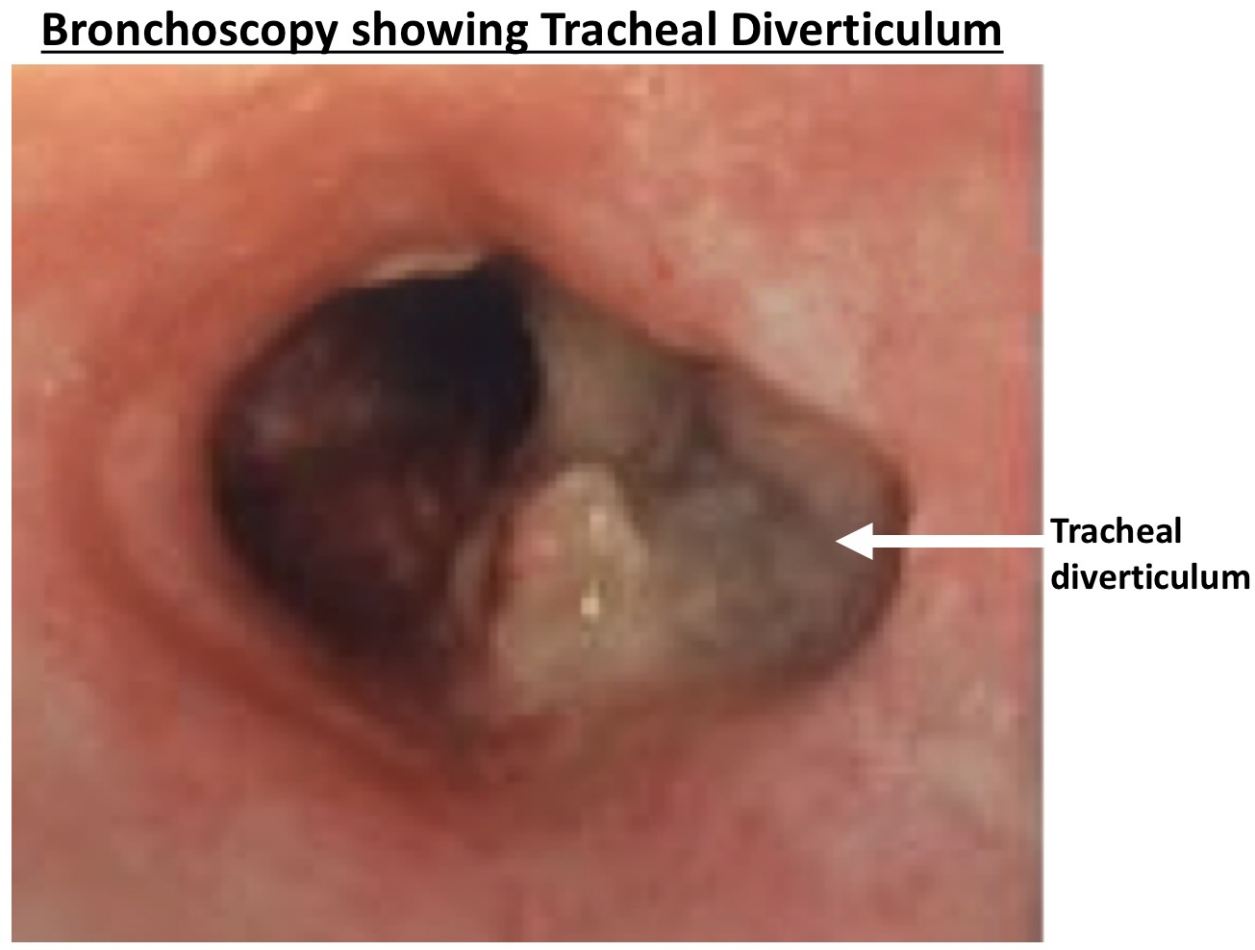
Bronchoscopy showing tracheal diverticulum, indicated by white arrow.

Six months after finishing her post-radiotherapy course of FOLFIRI-bevacizumab, she presented to our clinic with productive cough with thick sputum with occasional blood streaks for which she tried oral antibiotics with no improvement. We performed a CT scan, which showed a tracheal diverticulum in the region we had previously treated with radiotherapy (Figure 4). We performed bronchoscopy, which also revealed a large tracheal diverticulum (Figure 5).

**Figure 6:**
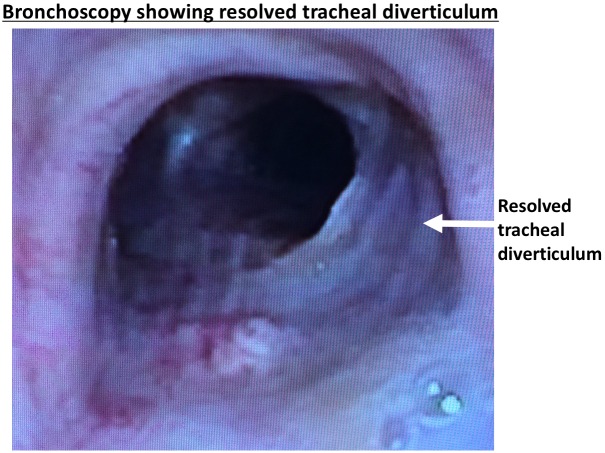
Bronchoscopy showing resolved tracheal diverticulum at most recent follow-up visit 15 months post-surgery.

We prescribed hyperbaric oxygen, pentoxifylline and vitamin E, which unfortunately did not improve her tracheal diverticulum per bronchoscopy performed three months later. We consulted our thoracic surgery colleagues who performed a serratus muscle flap repair of the tracheal diverticulum to prevent progression to tracheovascular fistula, as we were concerned the defect was extending toward the superior vena cava. The patient tolerated surgery well without complications, and was discharged home in stable condition on postoperative day 11. She has done well since then and continues to undergo routine monitoring with bronchoscopy and computed tomography (CT) imaging. Her most recent bronchoscopy, 15 months postsurgery, shows continued resolution of the tracheal diverticulum (Figure 6). In this case, due to expert surgical care we were able to avoid a potentially catastrophic complication.

## Discussion

Combination bevacizumab and radiotherapy has been shown to be effective for glioblastoma multiforme, but may be associated with significant adverse effects for treatment of other sites. This risk may be highest when treating mucosal sites. Stephens et al. showed increased rates of tracheoesophageal fistula within the radiation field in patients treated with VEGF inhibitors after hypofractionated radiotherapy adjacent to the esophagus [5]. Goodgame et al. reported the case of a 28-year-old man who developed tracheoesophageal fistula four months after thoracic radiotherapy while he was being treated with bevacizumab and chemotherapy [2]. Gore et al. reported the case of a 48-year-old man who developed tracheoesophageal fistula 21 months after thoracic radiotherapy while he was being treated with salvage bevacizumab and chemotherapy [6]. Spigel et al. reported the results of two independent phase II clinical trials in lung cancer where patients treated with radiotherapy also received induction, consolidation and maintenance bevacizumab and chemotherapy. Both trials were closed early due to high rates of tracheoesophageal fistula development [4]. Socinski et al. performed a phase I/II trial where patients received radiotherapy with induction, concurrent and consolidation bevacizumab, erlotinib and chemotherapy. The experimental regimen including bevacizumab and erlotinib showed a lack of efficacy, and led to high rates of grade III–IV esophagitis (29%) with one patient developing a tracheoesophageal fistula [3]. Finally, Pollom et al. described multiple cases of grade IIII–V toxicity when bevacizumab was administered before, after or concurrently with gastrointestinal stereotactic ablative radiotherapy (SABR) [7].

In this case, we administered highly conformal hypofractionated radiation therapy with definitive intent to an ultra-central right paratracheal nodal metastasis with the high dose region including a portion of the tracheal wall contralateral to the esophagus. Rather than tracheoesophageal fistula, we observed localized tracheal wall breakdown and diverticulum into the mediastinal fat after bevacizumab-containing systemic therapy was administered with a substantial time gap after the completion of radiation therapy. Because of concern of progression to potentially catastrophic fistula between the trachea and superior vena cava, a serratus muscle flap repair was performed with good success.

The hypofractionated radiation therapy regimen we used, 60 Gy in 15 fractions, was intensive because of definitive intent. By conventional linear-quadratic modeling, the equivalent dose in 2 Gy fractions would be 70 Gy for early/tumor effects (alpha/beta = 10) and 84 Gy for late effects (alpha/beta = 3). Even so, this falls within the range of what has been used safely in dose escalation studies of conventionally fractionated radiation therapy alone and chemoradiation for locally advanced lung cancer involving mediastinal nodes, and substantially less than stereotactic ablative radiotherapy regimens used safely for central and ultra-central (though generally not mediastinal) tumors such as 50 Gy in 4 fractions or 60 Gy in 8 fractions (156 Gy or 126 Gy EQD2 for late effects, respectively) [8-9]. We have previously demonstrated safety with the radiotherapy regimen used in this case (60 Gy in 15 fractions) when delivered with highly conformal technique in a phase I trial conducted by University of Texas Southwestern and Stanford in patients mostly with locally advanced disease [10], and confirmed this in our own subsequent experience. As such, tracheal wall breakdown after limited volume irradiation with this radiation regimen alone was highly unexpected.

It is unclear, however, when or if there is a safe way to combine bevacizumab with dose-intensive hypofractionated radiotherapy overlapping mucosal sites. Mucosal irradiation may preclude future administration of bevacizumab due to risk of toxicity, as bevacizumab may inhibit wound-healing after radiotherapy by diminishing angiogenesis to radiation-damaged tissue, which increases risk of treatment-related toxicity. We urge caution when considering dose-intensive radiotherapy to mucosal locations in association with VEGF inhibitors, and recommend close communication with medical oncologists to continue holding VEGF inhibitors after radiotherapy has been completed.

## Conclusions

We present a case of tracheal diverticulum following bevacizumab and paratracheal radiotherapy. We urge caution in treating patients with vascular endothelial growth factor (VEGF) inhibitors in the setting of hypofractionated radiotherapy involving the mucosa of tubular organs, even when these treatments are separated by months. Though data is limited as to the impact of sequence, this may be particularly an issue when VEGF inhibitors follow prior radiotherapy.
